# OperomeDB: A Database of Condition-Specific Transcription Units in Prokaryotic Genomes

**DOI:** 10.1155/2015/318217

**Published:** 2015-10-12

**Authors:** Kashish Chetal, Sarath Chandra Janga

**Affiliations:** ^1^Department of Biohealth Informatics, School of Informatics and Computing, Indiana University-Purdue University Indianapolis (IUPUI), 719 Indiana Avenue, Suite 319, Walker Plaza Building, Indianapolis, IN 46202, USA; ^2^Center for Computational Biology and Bioinformatics, Indiana University School of Medicine, 5021 Health Information and Translational Sciences (HITS), 410 West 10th Street, Indianapolis, IN 46202, USA; ^3^Department of Medical and Molecular Genetics, Indiana University School of Medicine, Medical Research and Library Building, 975 West Walnut Street, Indianapolis, IN 46202, USA

## Abstract

*Background*. In prokaryotic organisms, a substantial fraction of adjacent genes are organized into operons—codirectionally organized genes in prokaryotic genomes with the presence of a common promoter and terminator. Although several available operon databases provide information with varying levels of reliability, very few resources provide experimentally supported results. Therefore, we believe that the biological community could benefit from having a new operon prediction database with operons predicted using next-generation RNA-seq datasets. *Description*. We present operomeDB, a database which provides an ensemble of all the predicted operons for bacterial genomes using available RNA-sequencing datasets across a wide range of experimental conditions. Although several studies have recently confirmed that prokaryotic operon structure is dynamic with significant alterations across environmental and experimental conditions, there are no comprehensive databases for studying such variations across prokaryotic transcriptomes. Currently our database contains nine bacterial organisms and 168 transcriptomes for which we predicted operons. User interface is simple and easy to use, in terms of visualization, downloading, and querying of data. In addition, because of its ability to load custom datasets, users can also compare their datasets with publicly available transcriptomic data of an organism. *Conclusion*. OperomeDB as a database should not only aid experimental groups working on transcriptome analysis of specific organisms but also enable studies related to computational and comparative operomics.

## 1. Background

As the gap between the rate at which sequencing of complete genomes and the experimental characterization of transcriptional regulation in them increases, automated computational methods for unravelling the regulatory code are increasingly being sought after. Although accurate tools for gene identification encoded in a genome have been developed, our understanding on how the genes are expressed and regulated depends on our knowledge of how they are organized into operons-genes set that are cotranscribed to produce a single messenger RNA [1, 2]. Operons are the essential units of transcription in prokaryotic organisms and, as a result, identifying these structures is a main step in understanding transcriptional regulation. Knowing operon structure in a genome not only facilitates identifying sets of genes which are coregulated but also aids in other computational analyses, such as prediction of cis-regulatory elements, which often depend on accurate detection of operons. In addition, since operons often consist of genes that are related functionally and required by the cell for numerous biological processes, they are often good predictors of biological modules [3–5]. Therefore, deep understanding of operons will improve our knowledge of higher-order genomic associations and structures thereby expanding our understanding of various cellular networks composed of regulatory, structural, and functional pathways [5, 6]. Operons also provide insights into the cellular functions and also help in determining different experimental designs. In various recent high-throughput RNA-sequencing studies across a number of prokaryotic organisms, it has been convincingly shown that the structure of operons changes with the environmental conditions [7, 8], thus suggesting a need to the discovery and a better understanding of the transcriptional units originating from operons (predicted or otherwise) across experimental conditions in bacterial genomes. For all these reasons, the characterization of condition-specific transcription unit structure on a genomic scale is an important origin point for microbial functional genomics.

Several operon databases are currently available and provide information with varying levels of reliability and emphasis [9–13]. However, it is important to note that, traditionally, definitions of operons and transcription units are synonymously used for computational predictions, mainly because each operon was believed to encode for a single transcription unit (single polycistronic unit). However, emerging evidence from several RNA-sequencing studies supports a more complex model, with several operons in a genome encoding for multiple transcription units depending on the condition [7, 8]. Databases such as RegulonDB [13], which are based on manual curation of experimentally reported polycistronic transcripts identified in at least one experimental condition in the literature in* Escherichia coli *K-12, define an operon as the ensemble of all the transcription units in a given genome loci which results in the longest stretch of codirectional transcript. In such frameworks, each transcription unit is governed by a promoter and terminator identified in at least one condition. In contrast, working definition for computational prediction of operons across most studies simply assumes the longest possible polycistronic transcript in a genomic locus as an operon. These differences in the working definition indicate that the current prediction pipelines and databases for operon prediction are perfect in predicting condition-specific transcription units/operons in bacterial genomes. In OperonDB, Pertea et al. employed a method to find and analyze gene pairs that are located on the same strand of DNA in two or more bacterial genomes [10]. The computational algorithm used in this database locates operons structure in microbial genomes using a method published earlier by the authors [14]. OperonDB currently contains 1059 genomes with prediction sensitivity of 30%–50% in* Escherichia coli* [10]. DOOR (database for prokaryotic operons) is another database, which contains predicted operons for 675 sequenced prokaryotic genomes. It provides similarity scores between operons by which user can search for related operons in different organisms [9]. ProOpDB (prokaryotic operon database) predicts operons in more than 1200 prokaryotic genomes using a neural network based approach. It provides several options for retrieving operon information. In ProOpDB, users can also visualize operons in their genomic context and their nucleotide or amino acid sequences [11].

MicrobesOnline is another operon database, which facilitates the phylogenetic analysis of genes from microbial genomes [12]. In principle, this database has two functionalities: (1) user can build a phylogenetic tree for every gene family as well as a species tree in a tree-based browser to assist in gene annotation and in reconstructing their history of evolution and (2) by using its tool one can analyze microarray data to find genes which exhibit similar expression profiles in an organism which can subsequently be used for identifying regulatory motifs and seeing if they are conserved. User can also compare the organization of a protein domain with genes of interest in a browser [12]. Finally, as mentioned above, RegulonDB is a database [13], which is curated and designed for* Escherichia coli *K-12 to facilitate the prediction of its transcriptional regulatory network and operon organization across growth conditions. It also provides extensive information about the evolutionary conservation of a number of regulatory elements in* Escherichia coli *genome. The method used for predicting operons in RegulonDB has a maximum accuracy of 88% for the identification of pairs of adjacent genes in operon and it also identifies 75% of the known transcription units when used to predict the transcription organization of* Escherichia coli* [13]. However, there is not a single database present, which uses data from RNA-sequencing experiments to predict the transcription unit organization in a broad range of bacterial genomes in a condition-specific manner. In this study, we present operomeDB (http://sysbio.informatics.iupui.edu/operomeDB/#/) to address this gap—a database dedicated to the identification and visualization of transcriptional units from publicly available RNA-seq data in microbial genomes.

High-throughput sequencing platforms like Illumina, ABI, and Roche are used to quantify the expression levels of RNA in a condition-specific manner in bacterial genomes—frequently referred to as an RNA-seq experiment. Such high-throughput technologies have several advantages compared to traditionally used microarray platforms like a low background signal, large dynamic range of expression level, and possibility of detecting novel transcripts. There are different tools for detection, management, and analysis of the eukaryotic RNA-seq data; however relatively very few tools are available for the analysis and processing of RNA-seq data in prokaryotes. Rockhopper is an open source computational algorithm implemented for the analysis of bacterial RNA-seq data [15]. It supports different stages of RNA-seq analysis and datasets from different sequencing platforms. The algorithm performs several functions such as aligning the sequence reads to a genome, constructing transcriptome maps, calculating the abundance of transcripts, differential gene expression, and predicting transcription unit structure. It also has the ability to detect novel small RNAs, operons, and transcription start sites with a high accuracy in a transcriptome-specific manner [15].

Although there are many tools and software available for the visualization and exploration of next-generation sequencing datasets for eukaryotic organisms, there is a lack of proper genome browsers to visualize prokaryotic organisms and transcriptomes in particular. The size of data generated by RNA-sequencing methods is usually large and makes data visualization a challenging task. IGV (Integrative Genomic Viewer) is a visualization tool that can visualize large data sets very smoothly with the main aim of helping the researchers to visualize and explore the results [16]. The UCSC and Ensembl genome browsers are online tools that have been used to display different biological datasets, including genomic variants, expressed sequence tags, and functional genomic data with manually curated annotations [17, 18]. In this study, we used jBrowse to develop visualization of predicted transcription units for each RNA-seq dataset analyzed across genomes. jBrowse is an open source, portable, JavaScript based genome browser particularly suitable for prokaryotic genomes. The browser provides easy navigation of genome annotations on the web and has good track selection, zooming, panning, and navigation features [19].

We believe that biological community could benefit from having a new operon prediction database, which uses RNA-seq datasets to predict transcription units in a condition/transcriptome-specific manner. In our presented database (operomeDB) for bacterial genomes, we used an innovative approach to query operons. We predict operons for nine bacterial genomes for which at least few RNA-seq datasets are available in the public domain from the Sequence Read Archive (SRA) of NCBI [20]. We used Rockhopper [15] for the computational analysis of data. Using RNA-seq data for different bacterial genomes, the developed database, which to our knowledge is the largest of its kind to date, should facilitate to researchers navigating through operons predicted under different experimental conditions.

## 2. Construction and Content

### 2.1. Dataset Acquisition and Composition of the Datasets

We collected RNA-seq datasets for various bacterial species under a number of different conditions from the Sequence Read Archive (SRA) of NCBI [20] as described below. Descriptions of the bacterial genomes have been taken from Wikipedia and other cited resources as appropriate.

### 2.2. *Escherichia coli* K-12 MG1655

*Escherichia coli* is generally found in the colon and large intestine of the warm-blooded organisms. It belongs to a family of K-12 and B strains that is used in molecular biology for different experiments and also considered as a model organism. K-12 is the strain first confined from a sample of stool of the patient suffering from diphtheria. Different strains have emerged in years due to various treatment agents [21]. Expression profiling of wild type and SgrR mutant* E. coli* under aMG and 2-DG-induced strain was performed by Wadler and Vanderpool [22]. RNA-sequencing data available for illumine platform for this strain under 54 different conditions was analyzed using Rockhopper [15].

### 2.3. *Eggerthella lenta* DSM2243

*Eggerthella lenta* is an anaerobic, nonmotile, nonsporulating pathogenic gram-positive bacteria isolated from a rectal tumor. It is mostly found in blood and human intestine and can cause severe infections. Temperature favorable for growth of these bacteria is 37-degree Celsius [21]. Expression profiling study carried out for the generation of datasets is based on RNA-seq analysis of* Eggerthella lenta* cultured with or without digoxin. This dataset comprised 21 different transcriptomes in* Eggerthella lenta* DSM2243 strain from Haiser et al. [23].

### 2.4. *Campylobacter jejuni* RM1221

*Campylobacter* species are the prominent cause of gastroenteritis in countries on the path of development. An infection occurring due to* C. jejuni* is the most frequent preliminary cause for a neuromuscular paralysis, which is also known as Guillain-Barre syndrome. Healthy cattle and birds can carry* C. jejuni* [21]. For this study, data from Dugar et al. [24] where the authors did a comparative dRNA-seq analysis of multiple* Campylobacter jejuni* strains revealing conserved and specific transcriptional patterns to this strain was used. For 16 different conditions, RNA-seq data for* Campylobacter jejuni* RM1221 was obtained from this study [24].

### 2.5. *Clostridium beijerinckii* NCIMB 8052

*C. beijerinckii* NCIMB 8052 is anaerobic, motile, rod-shaped bacteria. The anatomy of the cell changes with the progression of growth cycle of the organism.* C. beijerinckii* species are present everywhere in nature and routinely segregated from soil samples [21]. Wang et al. carried out single-nucleotide resolution analysis of the transcriptomic structure of* Clostridium beijerinckii* NCIMB 8052 using RNA-seq technology [25]. This is comprised of expression quantification dataset for 6 different conditions in this organism [25].

### 2.6. *Clostridium difficile* 630

*C. difficile* is commonly found in water, air, human, and animal feces. Its genome reveals that the pathogen thrives in the gastrointestinal tract and some of its strains are more fatal than others. With the help of* C. difficile *genome we can understand the antimicrobial resistance and various treatment options available. After the sequencing of the whole genome, it was found from the whole genome that 11% of it consists of genetic elements such as conjugative transposons. These genetic elements contribute* Clostridium* with the genes subjected for their antimicrobial resistance, interaction to host, and surface structure production [26]. We used data from Fimlaid et al., where the authors conducted a global analysis of genes induced during sporulation of* Clostridium difficile* using Illumina HiSeq 1000 for 18 different conditions [27].

### 2.7. *Mycobacterium tuberculosis* H37rv

*Mycobacterium* is a causative agent of tuberculosis and has a waxy coating on its surface. Primary* Mycobacterium* affects respiratory system and lungs. H37rv strain of tuberculosis has 4 million base pairs with 3959 genes. The genome contains 250 genes that are involved in metabolism of fatty acids. Datasets for this genome are collected from experiments in which authors performed the high-resolution transcriptome and genome wide dynamics of RNA polymerase and NusA [28]. A total of 10 different transcriptomes were collected from this study for* Mycobacterium tuberculosis* [28].

### 2.8. *Salmonella enterica*  subsp. * enterica* Serovar Typhimurium str. 14028S

*Salmonella enterica* serovar is a subspecies of* S. enterica*, which are in the shape of rod, flagellated, aerobic, and gram-negative.* Salmonella* serovar can have many strains, which allows for accelerated increase in the total number of antigenically variable bacteria. In a study by Stringer et al. [29], authors used RNA-seq to conclude the effects of AraC and arabinose on RNA levels genomewide in* S. enterica*. Wild type or delta AraC mutant cells were developed in the presence and absence of 0.2% L-arabinose. The data for* Salmonella enterica* was collected for 8 different conditions [29].

### 2.9. *Sinorhizobium meliloti* 2011

*Sinorhizobium meliloti* is a nitrogen-fixing bacterium. Nitrogen fixation by* S. meliloti* is hampered by the plastic modifier bisphenol A. Dataset used in our database corresponded to a recent study where the authors performed RNA-sequencing of 18 samples corresponding to this bacteria in 3 different conditions [30]. For each condition, both short and long RNA fractions were analyzed, and three replicates per condition and per RNA fraction were performed. In this study next-generation annotation of prokaryotic genomes with Eugene-P was performed—applied to* Sinorhizobium meliloti* 2011 genome [30].

### 2.10. *Synechococcus elongatus* PCC 7942

*Synechococcus elongatus* are found in aquatic environments. They are called photosynthetic bacteria, as they are responsible for its production.* Synechococcus* consists of one circular chromosome and two plasmids. This particular strain contains a circular chromosome 2,700,000 bp long with GC content of 55%. For the generation of 17 datasets, three strains (7942, SE01, and SE02) were analyzed by Ruffing at two time points (100 h and 240 h) with three biological replicates [31].

### 2.11. Prediction of Operons Using Rockhopper

To predict transcription units (operons) in a condition/transcriptome-specific manner, we used Rockhopper, a computational algorithm which supports different stages of RNA-seq analysis for datasets originating from diverse sequencing platforms [15]. Rockhopper takes sequenced RNA reads as input in a number of formats including FASTQ, QSEQ, FASTA, SAM, and BAM files [15]. It allows the processing of next-generation RNA-seq data by permitting the user to specify different parameters to align sequence reads to a genome, such as number of mismatches allowed, orientation of mate-pair reads, and minimum seed length. For transcriptomic analysis in Rockhopper, some parameters specified include whether the dataset is strand specific, test for differential expression, prediction of operons and minimum expression of UTRs, and detection of ncRNAs. However, the authors recommend the use of default settings most of the time for best operon prediction performance and hence in this study we used the default parameters where possible [15]. Indeed, operon prediction by Rockhopper has been shown by the original authors to perform at ~90% accuracy when benchmarked against RegulonDB [13] and DOOR [9] databases. We also compared multigene operons predicted by Rockhopper for the nine genomes studied here using the RNA-seq data and the percentage of operons shared with the DOOR database predictions is shown in Table 1. We found that on average 93% of our predicted operons in a genome were independently confirmed to be operons by DOOR database suggesting the high quality of our operon predictions.

Each run of Rockhopper on a single RNA-seq dataset corresponding to a condition provides different files as output, such as summary file—which contains a summary analysis of successfully aligned reads to genomic regions, transcript file—which includes newly predicted transcripts, transcription start, and stop sites with expression levels. Finally, it provides operons file containing predicted operons in the condition. We ran Rockhopper in a batch mode to process and predict operons in each condition for each genomic dataset discussed above by selecting the appropriate reference sequence. We also ran operon prediction on the complete transcriptomic dataset for each genome to obtain a consensus set of operon predictions which was used to show as a reference operome (operon track) of the organism in jBrowse. In order to index the operons in our database, we numbered them by matching with the IDs of the predicted operons from DOOR database in order to easily know the novel operons. If our predicted operon shared at least one gene we gave the same operon ID as DOOR database and for operons which were not present in DOOR database we marked them as “NA.”

## 3. Utility and Discussion

### 3.1. Implementation and Interface

**Figure 1:**
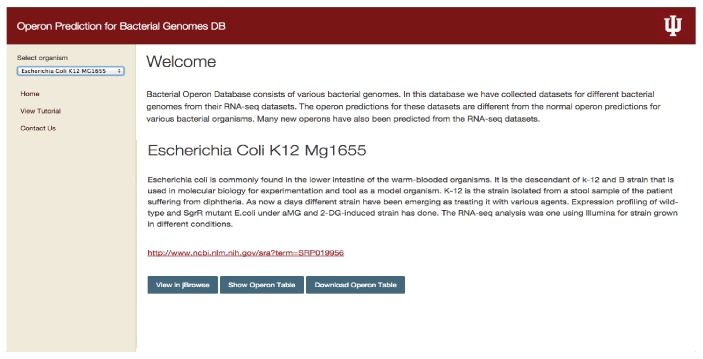
Web interface for operomeDB showing a screenshot of a selected bacterial genome to facilitate the browsing and download of predicted operons. The left panel of the webpage allows user to select an organism of interest. Once the user selects a bacterial organism the interface will provide information about the organism, experimental conditions under which RNA-seq datasets are available, and SRA link for experimental conditions and options to visualize in jBrowse, show operon table, and download the complete set of operon predictions across all the conditions as a table.

We developed an interface using HTML, CSS, and JavaScript and also incorporated jBrowse, a genome viewer to display different tracks for building operomeDB (http://sysbio.informatics.iupui.edu/operomeDB/#/) presented here. User interface of our database is shown in Figure 1 which allows the selection of an organism using a drop-down list. User can select an organism and selected bacterial genome information is displayed. There are multiple view options available for users, like viewing as a table of predicted operons, viewing operons using jBrowse, or downloading the predicted operons as a table (Figure 1). Clicking on the tab with the option of “view in jBrowse” will display data in jBrowse and user can view reference sequence of the genome, gene list in the particular bacterial genome, and a list of operons predicted. User can also select to show the operon data for different conditions from which RNA-seq datasets have been taken, with reference to their SRA IDs. Also, using SRA ID, user can search for a specific condition of each bacterial genome in the NCBI SRA database (http://www.ncbi.nlm.nih.gov/sra) [20].

**Figure 2:**
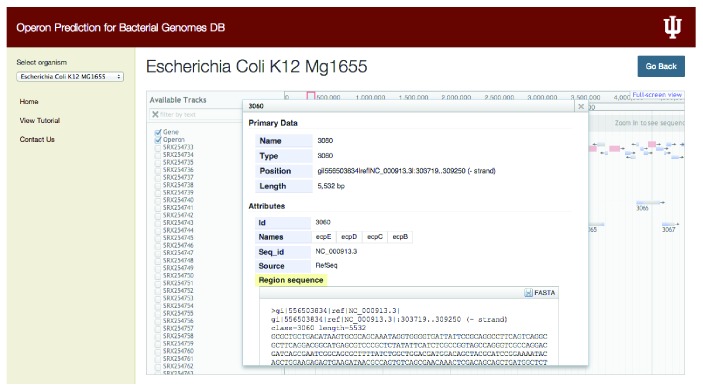
Screenshot showing the selection of an operon in the operons track. Highlighted is the ecpBCDE operon in* Escherichia coli *K-12 genome encoding for the membrane and fimbria formation proteins. This view provides the name (database generated ID), position, type, and length of the operon. It also gives information such as the number of genes present in the operon and sequence of the region for the selected operon.

For a selected operon or gene, jBrowse will provide detailed information such as genomic position of that particular operon, its length in base pairs (bp), and its primary attributes such as IDs, associated gene names, and source and sequence region in FASTA format (Figure 2). For a selected gene in the gene track, additional attributes such as Dbxref (reference id) and Gbkey (CDS, gene) are also displayed. Our database can generate a FASTA file containing user-specified operons and associated information and can be downloaded to the user's local computer for further analysis.

### 3.2. Visualization Using jBrowse

**Figure 3:**
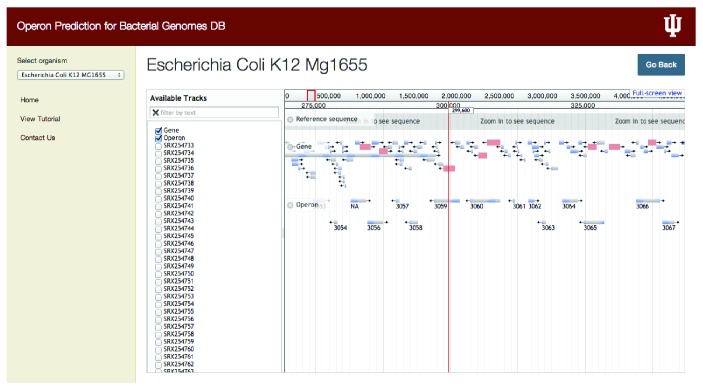
Snapshot of the jBrowse visualization showing the ensemble of all the operons predicted for a bacterial organism. User can select the reference sequence, genes present in the organism, and operons predicted from all the datasets and select the individual dataset to get the operons predicted for a particular experimental condition.

**Figure 4:**
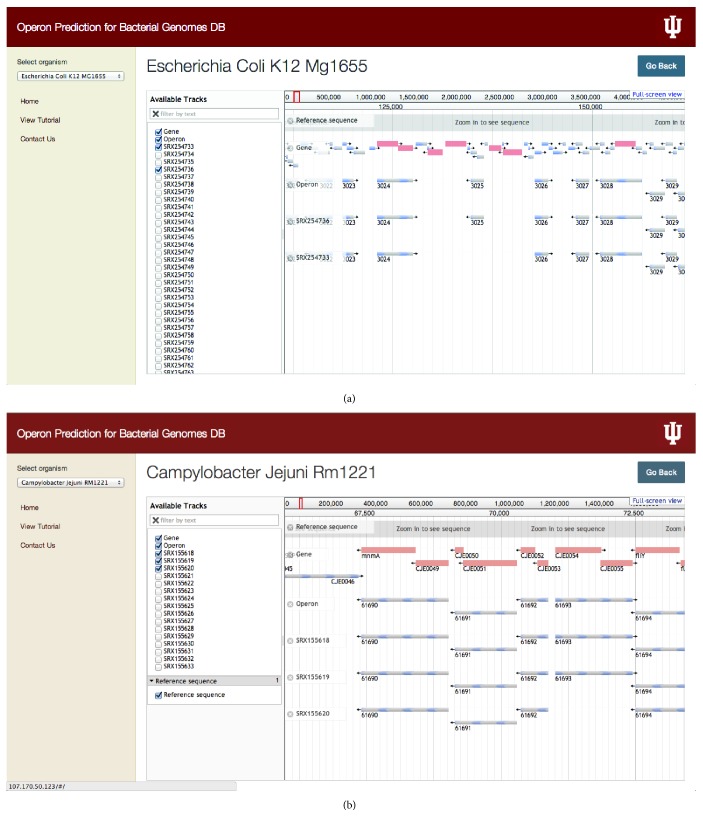
Presence and absence of operons for different experimental conditions in two different bacterial genomes. (a) For* Escherichia coli *K-12* MG1655* we have displayed the information for the missing operon “3025” in one of the experimental conditions SRX254733. (b) Another example is from* Campylobacter jejuni Rm1221* where we have displayed the information for the condition SRX155620 with missing operon “61693.”

In our database we incorporated jBrowse, which supports different file formats, and in our specific implementation we use FASTA files to display the reference sequence and BED, GFF, or BAM format files for displaying the list of genes and other discrete features such as operons [32]. User can select the particular operon and selecting that particular operon can display the length of the operon and genes constituting the specific operon as well as sequence for that particular operon (Figure 3). From jBrowse panel, user can also select any number of experimental conditions for which operon predictions using RNA-seq data are available, and it will display the operons for selected location. Users have the choice to display any number of tracks and visually compare them for downstream analysis. For instance, Figure 4 shows examples of predicted presence and absence of operons for different experimental conditions in* Escherichia coli *K-12* MG1655* and* Campylobacter jejuni RM1221*. It was found that certain operons in microbial genomes studied here were missing for a few experimental conditions. In our database we represent this variability of tracks with respect to the reference genome. For example, in* Escherichia coli *K-12* MG1655*, “3025” operon encoding for the genes speD (S-adenosylmethionine decarboxylase) and speE (spermidine synthase) was found to be missing in the experimental condition SRX254733 (Figure 4(a)). Such observations could be contributed due to the specific experimental condition which the experimentalists interested in the operon can explore further on a case-by-case basis. Another example shown in Figure 4(b) from the* Campylobacter jejuni RM1221* transcriptome also exhibits variability in operon organization. In this organism we found that “61693” operon (encoding for the poorly annotated ORFs CJE0054 and CJE0055) is missing in SRX155620. In our database multigene operons are predicted based on the cotranscription occurring in genes. Hence, the operons with a lack of occurrence of cotranscription would be identified as missing operons suggesting either a functional relevance of their absence or in few cases for very low abundant genes due to the lack of sequencing depth, in certain experimental conditions under study. We anticipate that, with increase in the depth and number of conditions for which RNA-seq datasets will become available, it will become easy to tease functionally important condition-specific transcription units via operomeDB.

Our system also allows a user to submit their own sequence in specific file format and database will display its contents as an additional track. Using options in jBrowse, user can easily upload their data files to jBrowse or paste URLs, where data is present to display its contents. Various file formats such as GFF3, BigWig, BAM index, BAM, and VCF are supported. User can also visualize and compare different tracks and hence analyze if there are similarities/dissimilarities between tracks. This feature will enable the comparison of new RNA-seq data for a given organism with already available public data for various experimental conditions available in operomeDB. Additionally, custom tracks will also enable comparison of operon tracks of different closely related organisms to study the variations in transcript architecture across the length of the genome.

In comparison to earlier resources of bacterial operons, our database offers high quality single-nucleotide resolution bacterial operon predictions based on high-throughput data sets.

## 4. Using the Database: An Example

**Figure 5:**
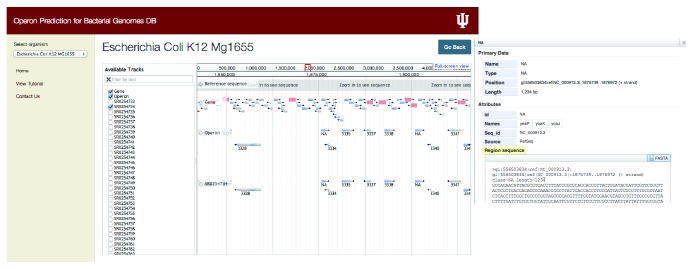
Operonic view showing a newly identified operon (yeaP-yoaK-yoaJ) in the genome of* Escherichia coli *K-12. In operomeDB, newly identified operons compared to other databases such as DOOR are marked as “NA” and user can further click on these to get the relevant information.

Below we provide an example illustrating the functionality of operomeDB. The presented example (Figure 5) is from* Escherichia coli *K-12* MG1655* genome for a newly identified three-gene operon yeaP-yoaK-yoaJ which has not been annotated in other databases such as DOOR [9] highlighting novel predicted operons that can be identified and visualized using our database.

1. A user can go to the main page of our Graphical User Interface (GUI) and click on “Select Organism” and then it will provide the list of the entire bacterial organisms present in the database. For instance, selecting the query genome as* Escherichia coli *K-12* MG1655* will display the page showing the operon predictions in various formats for* E. coli*.
2. On the query result page, it will display the information regarding* E. coli* and other possible options available. By clicking on the link “View in jBrowse” will enable the user to navigate the data via genome browser through different tracks.
3. In genome browser on the left panel user can select any number of available tracks and selected tracks will be displayed in the browser window. The user can now go through each track and query different operons predicted in our database.
4. Using the download button user can download the FASTA sequence file for a particular operon.
5. By selecting “file” option in upper panel, users can also upload/add their own sequence or dataset for visualization or comparison in the genome browser.
6. User can also look for predicted operons in each bacterial organism marked as “NA.” These are the operons that are newly predicted in our study compared to the DOOR operon database [9] (Figure 5).

## 5. Conclusion and Future Directions

Characterizing operon structures in a genome is one of the first and fundamental steps towards improving our understanding on transcriptional regulation in bacterial genomes. OperomeDB represents one of the first attempts to provide a comprehensive resource for operon structures in microbial genomes based on RNA-sequencing data, providing a one stop portal for understanding the genome organization in the context of transcriptional regulation in a condition-specific manner. OperomeDB as a database should not only aid experimental groups working on transcriptome analysis of specific organisms but also enable studies related to computational and comparative operomics.

In our study each SRA ID for which the operon prediction was performed corresponds to a different condition or perturbation to the cell in which RNA was sequenced to quantitate the expression levels of genes. Therefore, this database will be helpful for researchers not only to browse through each condition and analyze operons predicted for that particular condition but also to add their own new RNA-seq datasets corresponding to their experiments to uncover novel operon signatures specific to their condition of interest. Researchers can also compare operons predicted in our database with other databases under various conditions. Comparing operons under experimental and normal conditions will provide insight into the mechanism and effect of the particular condition on bacterial regulation at specific genomic loci. In the future, we will add more bacterial organisms with RNA-seq datasets to our database and we will also increase the number of datasets/conditions for already existing bacterial organisms in our database.

## 6. Availability and Requirements

OperomeDB can be accessed from the following URL: http://sysbio.informatics.iupui.edu/operomeDB/#/.

## Figures and Tables

**Table 1:** Table showing the percentage of multigene operons in the operomeDB found to be overlapping with the operon predictions for the corresponding genomes in the DOOR database.

Bacterial genome	Percentage (overlap)
*Clostridium difficile* 630	89
*Clostridium beijerinckii* NCIMB 8052	95
*Escherichia coli *k-12 mg1655	94
*Eggerthella lenta *dsm2243	95
*Mycobacterium tuberculosis* h37rv	91
*Salmonella enterica* subsp.	91
*Synechococcus elongatus* pcc7942	92
*Campylobacter jejuni *rm1221	97
